# Cell cycle regulation of proliferation versus differentiation in the central nervous system

**DOI:** 10.1007/s00441-014-1895-8

**Published:** 2014-05-25

**Authors:** Laura J. A. Hardwick, Fahad R. Ali, Roberta Azzarelli, Anna Philpott

**Affiliations:** 1 Department of Oncology, Hutchison/MRC Research Centre, University of Cambridge, Cambridge Biomedical Campus, Cambridge, CB2 0XZ UK; 2 Department of Clinical Neuroscience, John van Geest Centre for Brain Repair, University of Cambridge, Forvie Site, Cambridge, CB2 0PY UK

**Keywords:** Cell cycle, Differentiation, Neurogenesis, Proneural, Central nervous system

## Abstract

Formation of the central nervous system requires a period of extensive progenitor cell proliferation, accompanied or closely followed by differentiation; the balance between these two processes in various regions of the central nervous system gives rise to differential growth and cellular diversity. The correlation between cell cycle lengthening and differentiation has been reported across several types of cell lineage and from diverse model organisms, both in vivo and in vitro. Furthermore, different cell fates might be determined during different phases of the preceding cell cycle, indicating direct cell cycle influences on both early lineage commitment and terminal cell fate decisions. Significant advances have been made in the last decade and have revealed multi-directional interactions between the molecular machinery regulating the processes of cell proliferation and neuronal differentiation. Here, we first introduce the modes of proliferation in neural progenitor cells and summarise evidence linking cell cycle length and neuronal differentiation. Second, we describe the manner in which components of the cell cycle machinery can have additional and, sometimes, cell-cycle-independent roles in directly regulating neurogenesis. Finally, we discuss the way that differentiation factors, such as proneural bHLH proteins, can promote either progenitor maintenance or differentiation according to the cellular environment. These intricate connections contribute to precise coordination and the ultimate division versus differentiation decision.

## Introduction

During development of the central nervous system (CNS), a period of extensive proliferation is needed to generate the required number of progenitor cells for correct tissue and organ formation. This must be accompanied or closely followed by cell differentiation, in order to generate the range of functional neurons and glial cells at the correct time and place. Indeed, the temporal nature of neurogenesis and gliogenesis dictates that the processes of cell cycle progression and differentiation must be closely coordinated to generate a functioning CNS; too little proliferation might result in microcephaly and a loss of later-born cell types, whereas excessive cell division and/or a failure to differentiate would be equally detrimental and is characteristic of nervous system tumours. However, despite their central importance in developmental events, mechanisms ensuring precise coordination between cell division, cell cycle exit and differentiation have remained obscure until relatively recently. Here, we summarise the modes and dynamics of cell division within the developing CNS and describe mechanisms by which cell cycle regulators and differentiation factors can mutually influence and coordinate the division versus differentiation decision.

## Neural progenitor cell proliferation within the developing CNS

Neurogenesis proceeds through two major phases: an early phase of progenitor cell expansion and a neurogenic phase during which functional neurons are produced. Neural stem cells (NSCs) and their derivative precursor cells initially undergo symmetric proliferative cell divisions to expand the progenitor pool and later switch to asymmetric division followed by symmetric neurogenic division to generate the cellular diversity and differential growth within the various regions of the CNS (Zhong and Chia 2008; Götz and Huttner 2005). There are three main types of progenitor cells, namely the neuroepithelial cells, radial glial cells and intermediate (also known as basal) progenitors. Moreover, a new type of neural progenitor, the outer radial glial cell, has recently been discovered in primates and rodents (Fietz and Huttner 2011; Lui et al. 2011).

### Neuroepithelial cells

After closure of the neural tube, the epithelial lining of the ventricles becomes specialised, consisting of a single sheet of progenitor cells called neuroepithelial cells. These cells exhibit characteristic apico-basal movement of the nucleus in coordination with cell cycle progression, a phenomenon known as interkinetic nuclear migration (Sauer and Walker 1959). Importantly, just before mitosis, the nucleus moves towards the ventricular surface and the cell undergoes division at the most apical side, creating a pseudo-stratified appearance. Neuroepithelial cells undergo symmetrical cell divisions during the proliferative period to self-renew and expand the pool of progenitors (Zhong and Chia 2008).

### Radial glial cells

As neurogenesis proceeds, neuroepithelial cells transform into a different population of progenitor cells called radial glial cells. Collectively, these are known as apical progenitors on account of their apico-basal polarity, interkinetic nuclear migration and apically located cell division. Neuroepithelial and radial glial cells express identical markers such as the intermediate filament protein nestin and the transcription factor Pax6. However, radial glial cells are further characterised by the expression of astroglial markers, such as the glutamate transporter (GLAST) or the glial fibrillary acidic protein (GFAP) and the brain lipid binding protein (BLBP; Götz and Huttner 2005). During neurogenesis, radial glial cells divide asymmetrically both to self-renew and to maintain the pool of progenitors, and also to produce a neuronally committed daughter cell (Anthony et al. 2004; Hartfuss et al. 2001; Malatesta et al. 2000, 2003). The cell committed to the neuronal lineage becomes either a neuron (direct neurogenesis) or an intermediate progenitor that will undergo another division before leaving the proliferative areas (indirect neurogenesis).

### Intermediate or basal progenitors

Intermediate (also called basal) progenitors represent a second pool of neuronal progenitor cells. In contrast to radial glial cells, they exhibit multipolar processes that lack contact with the ventricular or pial surface and they are identified by the absence of Pax6 and by the expression of the transcription factor Tbr2/Eomes2, which marks early neuronal commitment (Englund et al. 2005). Basal progenitors undergo cell division away from the ventricular surface in more basal regions (Haubensak et al. 2004) and generally cycle only once or twice before undergoing symmetric neurogenic divisions to produce two post-mitotic neurons (Haubensak et al. 2004; Noctor et al. 2004).

### Outer radial glial cells

Outer radial glial cells are a new population of progenitors that are located in the outer part of the primate subventricular zone (SVZ; Hansen et al. 2010). These cells express radial glial markers, such as the transcription factor Pax6, the intermediate filament protein nestin and the astrocyte-specific marker GFAP, and yet they lack an apical contact and do not divide at the apical surface like radial glial cells. Instead, outer radial glial cells are located and divide in more basal regions, co-existing with the intermediate progenitor population, but are distinguished by their long cellular process that contacts the basal lamina and by their lack of expression of the basal progenitor marker Tbr2 (Fietz et al. 2010; Hansen et al. 2010).

## The cell cycle and neuronal differentiation

In general, one can characterise the development of the CNS as a phase of rapid progenitor expansion, followed by a gradual loss of proliferative capacity, concomitant with increasing cell fate restriction for given populations of progenitors. Ultimately, neuronal differentiation is accompanied by cell cycle exit into the quiescent G0 phase, although these two events can be artificially uncoupled (for example, Lacomme et al. 2012), thus demonstrating them to be potentially separable but highly coordinated processes. To understand the way that progenitor proliferation and differentiation are linked, we must first understand events that drive cell division.

**Fig. 1:**
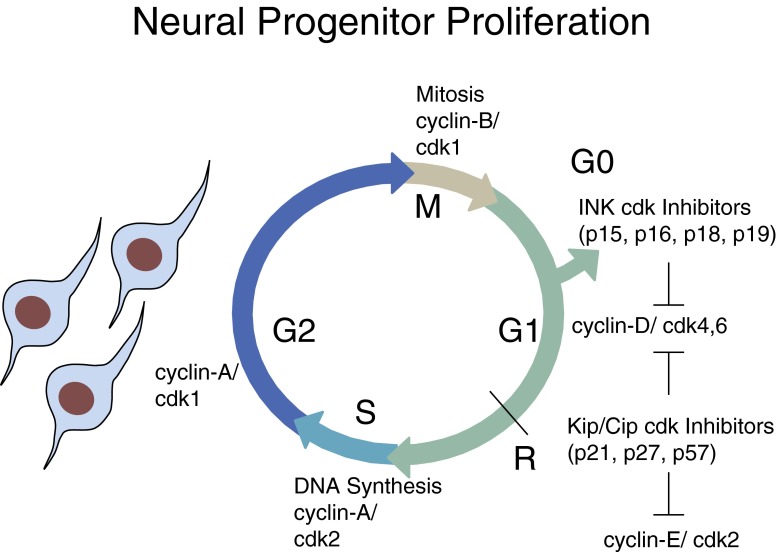
Representation of the cell cycle. Cell cycle progression is driven by the sequential activation and deactivation of a series of cyclin/cyclin-dependent kinase (*cdk*) complexes, which are further controlled by cyclin-dependent kinase inhibitors. The decision to divide or differentiate is typically made in early G1 phase, prior to the restriction point (*R*). Commitment to differentiate is accompanied by transition into G0 phase. Conversely, progression through R commits the cell to at least one more round of cell division

In summary, the cell cycle can be divided into four phases (Fig. 1): G1, S (DNA synthesis), G2 and M (mitosis). Unidirectional movement through these phases is driven by the activity of cyclin-dependent kinases (cdks) activated by specific cyclins. Cyclin-D/cdk4/6 effects passage through early G1, and cdk4-dependent phosphorylation of the retinoblastoma protein allows cells to pass through the G1 “restriction point”, after which they are committed to carry on through the cycle. Cells enter into S phase under the influence of cyclin-E/cdk2 and later cyclin-A/cdk2 and undergo semi-conservative DNA replication. G2 phase sees further cyclin-A and then cyclin-B accumulation, which is required for the activation of cdc2, which ultimately drives cells into mitotic division. Finally, the degradation of the mitotic cyclins by the Anaphase Promoting Complex (APC/C) leads to mitotic exit and re-entry into the next G1 phase. For key reviews, the reader is directed to Morgan (1995) and Nurse (2002) and, for a recent review, to Bertoli et al. (2013).

Cell cycle parameters are closely linked with cell fate specification and differentiation. The orderly generation and subsequent migration of newborn neurons gives rise to the six layers of the mammalian cortex, each layer containing neurons that share birth-date in addition to morphological and physiological characteristics. Classic cell transplantation experiments have revealed that the terminal laminar fate of deep-layer neurons is determined during the final S or G2 phase of the cell cycle preceding overt differentiation (McConnell 1995), thus indicating a cell cycle restriction for fate determination. More recent data has supported this idea: human embryonic stem cells (hESCs) in early G1 phase can only initiate differentiation into endoderm, whereas cells in late G1 are limited to neuroectodermal differentiation (Pauklin and Vallier 2013), indicating direct cell cycle influence on early lineage commitment in addition to terminal cell fate. Furthermore, as commitment to different fates occurs in different phases of the cell cycle, this suggests that context-dependent mechanisms are at work to link the cell cycle and differentiation (Ohnuma and Harris 2003).

## Cell cycle lengthening and neuronal differentiation

The correlation between differentiation and lengthening of the cell cycle has been independently reported across several different stem cell lineages (Lange and Calegari 2010). The developing murine cortex and in vitro cultures of human and mouse neural stem/progenitor cells have served as fruitful models in which to characterise cell cycle dynamics during progenitor cell proliferation and subsequent differentiation. Neural progenitor cells undergo an overall cell cycle lengthening immediately before neuronal differentiation and this is predominantly attributable to an extended G1 phase as progenitors switch from a mode of proliferative to neurogenic divisions (Calegari et al. 2005). Attention has focused on the importance of the G1 phase in determining the decision to divide or differentiate and nearly all G1 regulators have been shown to impact on neurogenesis in some way (for a review, see Hindley and Philpott 2012). Over the last decade, the mechanistic links between cell cycle length, particularly the length of G1, and the decision terminally to differentiate are becoming increasingly clear, with a number of studies seeking to manipulate cell cycle length and determining the consequences.

Early work demonstrated that the lengthening of the cell cycle by the down-regulation of cdk activity is necessary and sufficient for neuronal differentiation, both in vitro in PC12 cells (Dobashi et al. 2000) and in vivo in whole embryo mouse culture (Calegari and Huttner 2003). Because of the histogenic architecture of the cortex and retina, in which laminar identity is determined by birth-date (Desai and McConnell 2000), precocious cell cycle exit is also associated with changes in ultimate tissue architecture, either through a lack of later-born cell types in the cortex (Hatakeyama et al. 2004) or through altered cell fate specification in the retina (Ohnuma et al. 2002). Similar links between cell cycle length and differentiation have been observed in other species. For instance, the lengthening (but not necessarily arresting) of the cell cycle by overexpression of the cdk inhibitor (cdki) p27Xic1 can be enough to trigger precocious neuronal differentiation in developing *Xenopus* embryos (Vernon et al. 2003); p27Xic1 and the mammalian cdkis are discussed in detail below. However, because of the known multi-functionality of cdkis, experiments that simply overexpress cdkis cannot completely demonstrate that cell cycle length per se controls the propensity to differentiate. Instead, additional approaches to manipulate the expression of G1 regulators such as cyclins have been undertaken (Lange and Calegari 2010).

Acute overexpression of cyclin-D1/cdk4 by in utero electroporation in the mouse cortex at embryonic day 13.5 (E13.5) shortens the G1 phase by 30 % after 24 h and delays neurogenesis by enhancing proliferative divisions of basal progenitors. Conversely, acute knockdown of cyclin-D/cdk4 by RNA interference lengthens G1 by 20 % and increases the number of differentiated neurons by 40 % at 48 h but depletes the basal progenitor population for long-term neuronal output (Lange et al. 2009). Qualitatively similar changes are seen with the overexpression and knock-down of cyclin-D1 alone (Pilaz et al. 2009). Furthermore, this effect is conserved during adult neurogenesis in the hippocampus in which acute overexpression of cyclin-D/cdk4 by lentiviral injection results in a cell autonomous expansion of the progenitor pool and inhibition of neurogenesis when brains are analysed 1-3 weeks after injection (Artegiani et al. 2011). Similarly, the shortening of the cell cycle, achieved by the overexpression of cyclin-A2/cdk2 in developing *Xenopus* embryos, results in a delay of neuronal, but not muscle differentiation (Richard-Parpaillon et al. 2004).

A relationship between cell cycle length and differentiation is also observed in ESCs and NSCs in culture. Overexpression of cyclin-E in pluripotent mouse ESCs can protect against the pro-differentiation effects of transient deprivation of leucocyte inhibitory factor in the culture conditions (Coronado et al. 2013), whereas treatment of adult NSCs with a cdk4 inhibitor promotes differentiation under both self-renewing and induced differentiation culture conditions (Roccio et al. 2013).

Taken together, these results have led to the “cell cycle length hypothesis”, which postulates that the length of G1 is a critical determinant of differentiation (Calegari and Huttner 2003); a G1 phase beyond a certain threshold length is required for the sufficient accumulation and action of fate-determining factors that will then drive differentiation. However, if G1 phase is shorter than this threshold, differentiation will not occur and passage into S and G2 is not permissive for the differentiation signal to be executed. This model is also consistent with the cell-cycle-dependent regulation of the activity of key proneural basic helix-loop-helix (bHLH) transcription factors that control neuronal differentiation (see below).

It is interesting to view this model in the light of the recent data indicating that hESCs show differential susceptibility to lineage specification signals depending on cell cycle phase (Pauklin and Vallier 2013), whereas ESCs show changes in global epigenetic marks depending on their position in the cell cycle (Singh et al. 2013). Thus, the relative importance of the respective phases of the cell cycle might vary depending on the cell type and the nature of the exogenous determination signals. This is also consistent with recent work in chick spinal cord progenitor cells (Peco et al. 2012). Spatial patterning and neural induction in the spinal cord are regulated by morphogen gradients of Sonic hedgehog (Shh) and bone morphogenetic protein (BMP) signalling (Briscoe and Ericson 2001). Shh additionally upregulates CDC25B, a cell-cycle-associated phosphatase that becomes co-expressed with CDC25A in cycling progenitor cells at the onset of neurogenesis. Concomitant with the initiation of differentiation, the CDC25B-expressing progenitors also display a shortened G2 phase, which the authors suggest may limit cell sensitivity to Notch or Wnt signals that would otherwise promote progenitor maintenance (Peco et al. 2012). This is of interest, not only as it opens the debate as to the importance of the G2 phase for neurogenesis, but it also exemplifies a neurogenic function for a positive cell cycle regulator.

## Direct regulation of neurogenesis by cell cycle components

### Cyclins and cdks

Components of the cell cycle machinery are not uniformly distributed during neurogenesis. Indeed, many cell cycle components are expressed in specific tissues and developmental stages in a manner that cannot be solely accounted for by differences in cell cycle rates (Vernon and Philpott 2003). This suggests that these cell cycle regulators have a more direct role in the regulation of neurogenesis and that this role may or may not be linked to their ability to alter cell cycle parameters. Analysis of knock-out mouse models can be complicated by extensive redundancy and facultative compensation between cell cycle components; for example, NSCs from knock-out mice lacking both cdk2 and cdk4 are still able to replicate in culture, because of the compensatory activity of cdk1 and the up-regulation of cdk6 and cyclin-D1 (Lim and Kaldis 2012). However, these knock-out models can additionally reveal overt or microstructural neuronal phenotypes indicating tissue-specific functions and potentially cell-cycle-independent roles for specific cell cycle components. The cortical plate in the aforementioned cdk2 and cdk4 double-knock-out mice is reduced by over 46 % at E13.5, with a corresponding severe reduction in basal progenitor population. Although knock-out cells retain the ability to replicate, the lengthening of the G1 phase because of the lack of cdk2 and cdk4 results in premature symmetric neurogenic divisions (see above) that depletes the basal progenitor population, reducing the long-term neuronal output (Lim and Kaldis 2012). Similarly, based on the phenotypic analysis of knock-out mice, cdk6 has been ascribed a unique role in adult neurogenesis in the dentate gyrus (Beukelaers et al. 2011).

D type cyclins also show a differential expression pattern in the developing cortex, with cyclin-D1 and D2 predominantly being expressed in the ventricular zone (VZ) and SVZ, respectively (Glickstein et al. 2009). In addition, cyclin-D2 shows a distinct pattern of subcellular distribution, such that cyclin-D2 is asymmetrically inherited only in one daughter cell, which then will re-enter the cell-cycle (Tsunekawa and Osumi 2012). Whereas cyclin-D2 can compensate for cyclin-D1 deficiency in the VZ, the basal progenitor population in the SVZ shows a cyclin-D2 dependence for proliferation, and the corresponding knock-out model results in microcephaly (Glickstein et al. 2009). Furthermore, cerebellar progenitor cells are also dependent on cyclin-D2 for the postnatal proliferation phase that is required for the formation of molecular layer interneurons (Leto et al. 2011). Interestingly, there appears to be a more consistent role for the unique pro-proliferative functions of cyclin-D2, whereas cyclin-D1 has been associated with unique pro-differentiation functions. By gain of function and loss of function studies in the spinal cord, Lukaszewicz and Anderson (2011) have revealed that cyclin-D1 has a cell-cycle-independent role in the differentiation of motor neurons. Moreover, cyclin-D1 can have a direct role in the control of gene expression in the developing retina, apparently by promoter binding and recruitment of epigenetic modifiers in a cdk-independent manner (Bienvenu et al. 2010).

Not all cdks promote cell cycle progression; cdk5 is an unusual member of the cdk family in that it is not activated by cyclins but instead by the binding of an unrelated protein p35. Moreover, the expression of cdk5 is not associated with cycling cells but, instead, is highly expressed in differentiating and mature neurons in which it plays diverse roles such as promoting neuronal migration, neurite extension and synaptogenesis, as reviewed in Dhariwala and Rajadhyaksha (2008) and Dhavan and Tsai (2001). An intriguing finding is that cyclin-E expression is retained in terminally differentiated neurons in which it has a cell-cycle-independent function to facilitate synapse formation, through the binding and sequestering of cdk5 in a kinase-inactive complex (Odajima et al. 2011). Studies such as these demonstrate the benefit of further examination of cell cycle regulators that are expressed in a manner inconsistent with their known roles, as this may well reveal new and unexpected functions.

### Cdk inhibitors

Cdkis of the Kip/Cip family, namely p21Cip1, p27Kip1 and p57Kip2, have recognised roles in regulating the activities of cyclin-cdk complexes containing cyclins D, E and A (Sherr and Roberts 1999). These cdkis are also ideally placed both spatially and temporally to coordinate both cell cycle exit and neuronal differentiation during development (Nguyen et al. 2006a), and an increasing body of literature exists to demonstrate various cell-cycle-independent and non-redundant functions of these cdkis during neuronal specification and differentiation, maturation and migration (for reviews, see Ohnuma et al. 2001; Cremisi et al. 2003; Bally-Cuif and Hammerschmidt 2003; Tury et al. 2012).

#### Cdki function during neuronal specification and differentiation

The analysis of cdki knock-out mouse models can be complicated by redundancy between the mammalian Kip/Cip family proteins, and early studies utilised the developing *Xenopus* embryo, with a single prominent p27Xic1 homologue at these stages, to demonstrate the role of cdkis during neurogenesis (Vernon et al. 2003; Carruthers et al. 2003). Overexpression of p27Xic1 at a high level in the developing *Xenopus* embryo leads to cell cycle arrest and massive cell death. However, the expression of lower levels of p27Xic1 results in the precocious differentiation of neural plate progenitors into primary neurons (Vernon et al. 2003). Furthermore, the depletion of p27Xic1 impairs the formation of endogenous primary neurons and results in the accumulation of progenitor cells that are unable to transition to differentiation (Vernon et al. 2003; Carruthers et al. 2003).

Functions of mammalian p27Kip1 in regulating proliferation are readily demonstrated by the p27-knock-out mouse model, which exhibits systemic hyperplasia and increased cellularity in many tissues and organs (Fero et al. 1996; Kiyokawa et al. 1996; Nakayama et al. 1996). Loss of function approaches also identify roles of p27Kip1 specifically in the developing CNS. For example, p27Kip1 functions in regulating the proliferation of transit amplifying progenitors in the developing SVZ (Doetsch et al. 2002; Mairet-Coello et al. 2012) and in the postnatal neuronal cells that contribute to the olfactory bulb (Li et al. 2009). The thickened cerebral cortex in p27Kip1-null mice results from the expansion of projection neurons in layers II-IV and GABAergic interneurons in layers V and VI; based on birth date, this indicates altered neuron production during mid- to late-term neurogenesis, which is consistent with the time at which p27Kip1 mRNA peaks in these progenitor cells (Goto et al. 2004). Interestingly, however, cell cycle length and G1 phase are not found to be significantly altered by the loss of p27Kip1 function (Goto et al. 2004; Tarui et al. 2005).

In addition to promoting generic differentiation, p27Xic1 can also influence cell fate within the developing *Xenopus* retina, and overexpression of p27Xic1 leads to both premature cell cycle exit and conversion of retinal progenitor cells into Müller glial cells (Ohnuma et al. 1999). Intriguingly, the overexpression of *Xenopus* p27Xic1 in the mammalian retina produces similar effects with increased numbers of Muller glial cells, but overexpression of mammalian p27Kip1 does not directly alter retinal cell fate (Dyer and Cepko 2001) indicating a mechanistic difference between these two orthologues. P57Kip2 has, however, been ascribed a specific role in the specification of amacrine neurons in the mammalian retina (Dyer and Cepko 2000), and so, as *Xenopus* has a single Kip/Cip homologue at this stage of development, a cell fate function in the retina might be analogous to p57Kip2 in this respect.

In support of this idea, expression patterns and genetic manipulation strategies have revealed specific roles for p27Kip1 and p57Kip2 in regulating neurogenesis in various subsets of progenitor cells. In the cortex, p57Kip2 is more abundantly expressed during early corticogenesis (Tury et al. 2011) and regulates progenitors in both the VZ and SVZ, specifically regulating neuron production for layers V-VI (Mairet-Coello et al. 2012). P27Kip1 expression is highest at later stages (Tury et al. 2011) and regulates SVZ progenitors contributing neurons to layers II-V (Mairet-Coello et al. 2012). Furthermore, although both p27Kip1 and p57Kip2 act as modular proteins, the domains required for specific non-cell-cycle functions differ between the two, possibly indicating a different mechanism of action (Tury et al. 2011). Overexpression experiments indicate that p57Kip2 is more effective than p27Kip1 in inducing neuronal differentiation (Tury et al. 2011) but knockdown of p57kip2 by RNA interference does not reveal any defects in the differentiation of neurons (Itoh et al. 2007). An increased understanding of the mechanistic action of these two cdkis will help to elucidate their various shared or non-redundant functions (see below). Both p27Kip1 (Nguyen et al. 2006b; Kawauchi et al. 2006) and p57Kip2 (Tury et al. 2011; Itoh et al. 2007) also promote neuronal migration, demonstrating additional functions in neuronal maturation.

Furthermore, cdkis have been implicated in the differentiation of glial cells in the nervous system, and p27Kip1 and p21Cip1 might serve functionally separate and non-redundant roles during oligodendrocyte differentiation. Classic studies show that p27Kip1 gradually accumulates in oligodendrocyte progenitors and forms a component of both the timer and effector mechanisms that determine a limited number of cell divisions before terminal differentiation (for a review, see Durand and Raff 2000). However, both p27Kip1 and p21Cip1 are required for oligodendrocyte differentiation; whereas p27Kip1 is required for proper cell cycle withdrawal, p21Cip1 is instead required for the onset of differentiation, independently of its function as a cdki (Zezula et al. 2001). Similarly, p57Kip2 levels increase over time in oligodendrocyte precursors and form part of the intrinsic timer mechanism to regulate the number of divisions before differentiation (Dugas et al. 2007).

#### Mechanisms of cell-cycle-independent cdki function

Whereas p27Xic1 overexpression in *Xenopus* clearly slows the cell cycle, its ability to induce ectopic neuronal differentiation notably localises to an N-terminal domain of the molecule and is independent of its role as a cdki, being possibly related to an ability to stabilise proneural protein Neurogenin2 (Vernon et al. 2003). The separation of functions to distinct structural domains is also conserved in mammalian p27Kip1, together with an ability to interact with and stabilise proneural protein Ngn2 to promote neuronal differentiation in the mammalian brain (Nguyen et al. 2006b). Consistent with this, p27Kip1 knockout cells from the adult mouse SVZ region have reduced neuronal output attributable to the enhanced degradation of proneural proteins via the proteasome (Gil-Perotin et al. 2011).

Cdkis can regulate gene expression either directly or by regulating transcription factor function. The ability of p27Xic1/Kip1 to stabilise proneural protein Ngn2 has been discussed above (Vernon et al. 2003; Nguyen et al. 2006b). P27Kip1 has also recently been shown to be part of a repressive complex on the Sox2 promoter (Li et al. 2012) indicating an active role in suppressing progenitor maintenance while simultaneously promoting differentiation. Similarly, p57Kip2 can interact with nuclear receptor Nurr1 in a cell-cycle-independent manner to promote a dopaminergic fate of midbrain neurons (Joseph et al. 2003). However, p57Kip2 can also repress the transcriptional activity of proneural proteins such as Ascl1 and NeuroD, which might be important to enable proper glial cell differentiation (Joseph et al. 2009). Thus, p57Kip2 might regulate neurogenesis and gliogenesis in a context-dependent manner (Tury et al. 2012). These studies illustrate nicely the multi-functionality of cell cycle regulators that allows the precise coordination of the many parameters that accompany the transition from progenitor to differentiating neuron. We undoubtedly have some way to go to identify all the ways that this crucial class of cell cycle regulators is able to influence the differentiation and maturation process.

### Retinoblastoma protein

The retinoblastoma (Rb) protein is another key regulator of G1, traditionally recognised for its central role in the G1 restriction point to control the commitment of the cell to a further round of replication (for a recent review, see Dick and Rubin 2013). The role of Rb in aspects of neuronal differentiation and survival has been an evolving story since the early 1990s. Generation of the Rb-null mouse revealed an embryonic lethal phenotype; death occurs between E14 and E15, with embryos displaying severe developmental defects that notably affect the nervous and haematopoietic systems (Clarke et al. 1992; Jacks et al. 1992; Lee et al. 1992). Ectopic mitoses are observed throughout the CNS and peripheral nervous system, and this is accompanied by massive cell death, particularly in the hindbrain and sensory ganglia (Lee et al. 1992, 1994). Mechanistically, this has been associated with abnormal S phase entry attributable to elevated E2F DNA-binding activity and with activation of p53-mediated apoptosis in the CNS (Macleod et al. 1996).

Subsequent work has demonstrated, however, that this severe null-phenotype results indirectly from placental insufficiency (Wu et al. 2003). Loss of Rb leads to extensive proliferation of the trophoblast cells, which compromises placental vasculature; sustenance of Rb-null embryos with a wild-type placenta can prevent most of the neurological and haematopoietic abnormalities otherwise observed (Wu et al. 2003). Thus, mass apoptosis in the null model may be triggered indirectly, for example, by hypoxia, rather than as a direct effect of Rb loss in neuronal cells, and this is supported by chimeric studies in mice expressing both wild-type and Rb-null cells. Similar to Rb-null embryos, the brains of mid-gestation chimeras show extensive ectopic S phase entry but, in contrast to the Rb-null model, Rb-deficient cells in chimeras are still able to survive and differentiate into neurons, albeit arrested at the G2 phase of the cell cycle. Additionally, adult brains show an overall normal architecture (Lipinski et al. 2001). Tissue-specific knock-out of Rb in the developing telencephalon also results in ectopic cell divisions without widespread apoptosis, and the ectopically dividing cells are able to express early neuronal markers (Ferguson et al. 2002). Furthermore, conditional mutant cortices are still able to generate the full repertoire of cortical projection neurons and interneurons, despite the abnormal terminal mitosis (Ferguson et al. 2005). This is consistent with observations during *Xenopus* development in which Rb is not absolutely required for neuronal differentiation and, indeed, Rb remains hyper-phosphorylated, and therefore presumably inactive, well into tadpole stages, even though extensive neuronal differentiation has occurred by then (Cosgrove and Philpott 2007).

A refined model can therefore be presented whereby Rb functions cell-autonomously to regulate the cell cycle, but largely indirectly in neuronal differentiation and survival, with only a few specific cell types displaying a selective Rb requirement (Lipinski et al. 2001). For example, within the nervous system, a selective cell-autonomous role for Rb is described for the survival of cerebellar Purkinje neurons (Lipinski et al. 2001) and cerebral Cajal-Reizius neurons (Ferguson et al. 2005). Consistent with this model, even in the presence of a wild-type placenta, the rescued Rb-null mice still die perinatally with defective skeletal myogenesis and excessive apoptosis in the lens of the eye (de Bruin et al. 2003), indicating the presence of tissues with an absolute requirement for Rb for proper development; similar ocular defects are also observed in the chimeric embryos described above (Lipinski et al. 2001).

Additionally, Rb might function subsequent to the neuronal specification and early differentiation stage, instead influencing aspects of neuronal maturation and migration. Loss of Rb function in the developing mouse cortex results in impaired radial migration of early born dorsal telencephalon neurons and defective tangential migration of GABAergic interneurons from the ventral telecephalon (Ferguson et al. 2005). Mechanistically, Rb represses the E2F-mediated transcription of a chemotropic ligand receptor, neogenin, that otherwise leads to aberrant migration and adhesion (Andrusiak et al. 2011).

More recent work has focused on the mechanistic basis of Rb function in specific cell types. In addition to its established function of regulating the E2F transcription factor family, which is crucial for driving the expression of a number of vital cell cycle progression factors (such as cyclin-E and cdc2), Rb has been shown to regulate several aspects of neurogenesis directly and might interact directly with bHLH or HLH proteins, as reviewed in Ferguson and Slack (2001). For example, by forming part of a complex with NeuroD1 and orphan nuclear receptor RGF1-B, Rb has been shown to enhance the transcription activity of the bHLH protein NeuroD1 at the POMC promoter in the pituitary gland (Batsché et al. 2005). Interestingly, it is in the context of pituitary tumorigenesis that the interaction between Rb and the bHLH inhibitor ID (inhibitor of differentiation) proteins has been described (Lasorella et al. 2000, 2005). This interaction prevents ID activity and promotes differentiation, further supporting a tumour suppressor activity of Rb that goes beyond its classic role in the inhibition of cell cycle progression. Even within the Rb-E2F pathway, our comprehension of both cell-cycle- and non-cell-cycle-associated functions is evolving. For example, within the retina, Rb limits proliferation through the inhibition of E2F1, and yet Rb independently regulates the differentiation of cholinergic starburst amacrine cells (SACs) through E2F3a, without influence on cell cycle kinetics (Chen et al. 2007). Although our understanding has progressed from the early Rb-null models, we still do not know whether the phosphorylation status of Rb affects its neurogenic activity and, thus, to what extent the roles of Rb in neuronal differentiation are cell-cycle-dependent.

### Geminin

Geminin is a further example of an important factor with separable and conserved roles in the cell cycle and neurogenesis, as reviewed in Seo and Kroll (2006) and Luo and Kessel (2004). Geminin has previously been associated with the regulation of DNA replication licensing; accumulation during S phase enables Geminin to inhibit the re-initiation of DNA synthesis and thereby to prevent two rounds of DNA replication within each cell cycle. Towards the end of mitosis, Geminin is degraded together with cyclin-B by APC/C; this allows a new round of DNA replication to be initiated in the following S phase (McGarry and Kirschner 1998). Concurrent with the characterisation of its role in replication, Geminin has also independently been identified as a neuralising protein in *Xenopus* embryo ectoderm and is highly expressed at the onset of gastrulation in the area that later forms the neural plate (Kroll et al. 1998).

Geminin promotes early neural lineage specification from pluripotent progenitor cells but then keeps these progenitors in a proliferative and neural-primed state prior to subsequent differentiation (Seo and Kroll 2006; Luo and Kessel 2004). In this respect, Geminin participates in the dynamic equilibrium between proliferation and differentiation in neuronal progenitors, a function that appears to involve competitive interactions with various transcription factors and chromatin remodelling complexes (Luo and Kessel 2004; Pitulescu et al. 2005). In *Xenopus* embryos, this role in early neural specification is correlated with an ability to suppress BMP4 expression within the presumptive region of the neuroectoderm and with the up-regulation of the expression of proneural genes (Kroll et al. 1998). More recent work has revealed that Geminin additionally establishes an epigenetic state that favours the adoption of neuroectodermal fate, resisting sub-threshold stimuli for alternative lineage fates (Lim et al. 2011). This is dependent upon Polycomb repressor function (Lim et al. 2011) and is consistent with studies in mouse ESCs, whereby Geminin can inhibit mesendodermal fate specification through both a reduction in Wnt signalling and enhanced Polycomb repressor activity at key mesendodermal genes (Caronna et al. 2013).

Despite its key role in promoting neuronal lineage specification, Geminin subsequently maintains the neural progenitor state and resists premature neuronal differentiation in both *Xenopus* and mammalian cells (Seo et al. 2005a). Consistent with a role in epigenetic regulation, Geminin promotes a bivalent chromatin state in mammalian cells, at key transcription factor genes that promote neurogenesis; the presence of both activating and repressive histone modifications enables these genes to be repressed but poised for activation (Yellajoshyula et al. 2012). In the *Xenopus* model, Geminin interacts and inhibits Brg1, the catalytic subunit of a SWI/SNF chromatin-remodelling complex that is required for the transcriptional activity of bHLH proneural proteins during neurogenesis (Seo et al. 2005b). In this way, Geminin prevents the premature activation of the neurogenic cascade downstream of Ngn2 and NeuroD and regulates the timing of neurogenesis; activation of bHLH target genes occurs as Geminin levels decrease at the onset of neurogenesis (Seo et al. 2005a). Interestingly, Geminin has also been associated with a role in the long-term repression of neuronal genes in non-neuronal cells, acting in parallel to the established REST/NRSF repressor complex and this might also contribute to preventing premature neuronal gene expression in the developing mouse CNS (Kim et al. 2006).

An emerging and recurrent theme is the ability of Geminin to competitively inhibit transcription factor activity. For example, Geminin has a bidirectional inhibitory interaction with Six3, a homeodomain protein involved in eye formation, and a similar relationship is observed with patterning factor Hox homeodomain proteins (Pitulescu et al. 2005). Whereas Geminin can inhibit Hox protein function by associating with the Hox-Polycomb multi-protein complex, this interaction additionally prevents Geminin from regulating Cdt1 during DNA replication licensing (Luo et al. 2004).

The ability of Geminin to participate in many independent functions is facilitated by the distinct structural domains within the protein (for a review, see Pitulescu et al. 2005). For example, cell-cycle-associated roles reside in the C terminus, whereas neuralisation functions require the N terminus (Kroll et al. 1998; Pitulescu et al. 2005). Further characterisation of these multiple protein interactions will no doubt improve our understanding of the mechanisms by which this key protein influences the balance between proliferation and differentiation.

## Neuronal transcription factors that alter cell cycle dynamics

Just as components of the cell cycle machinery have a direct role in many aspects of neurogenesis, a number of neuronal transcription factors directly regulate the cell cycle. Karsten et al. (2003) have provided in vitro microarray data to characterise changes in global gene expression profiles during the transition from proliferating NSCs to differentiating neuronal cultures. By focusing on proteins that were specifically enriched in vivo in the proliferating neuroepithelial zones, as opposed to those with more widespread expression in replicating tissues, they have identified components of signal transduction and metabolic paths in addition to specific transcription factors that might form part of a gene network to coordinate cell cycle and cell fate events. Transcription factors such as Sox3 and FoxM1 are enriched in the germinal zone progenitors (Karsten et al. 2003) and belong to families of transcription factors with multiple complex roles during neurogenesis (Hindley and Philpott 2012).

The Forkhead transcription factor FoxM1 has a conserved expression in neural progenitor cells (Ueno et al. 2008; Karsten et al. 2003; Laoukili et al. 2005), with levels increasing from the start of S phase and remaining high throughout mitosis. Loss of function by gene knock-out produces an embryonic lethal phenotype with cells displaying extensive polyploidy and multiple cell cycle defects. Mechanistically, FoxM1 is associated with the transcriptional regulation of a cluster of genes required for the correct execution of mitosis; this includes cyclin-B1 for entry into mitosis, and CENP-F, which functions during the spindle assembly checkpoint (Laoukili et al. 2005). Additionally, this Fox-M1-dependent cell division is required, although not sufficient, for neuronal differentiation in the early *Xenopus* embryo. Neural induction occurs during gastrulation stages of embryo development and requires the inhibition of BMP signalling with the induction of cell proliferation in the neuroectoderm through the activation of FoxM1. Neuronal differentiation and cell cycle withdrawal occurs shortly afterwards but, in the absence of FoxM1, the formation of primary neurons is markedly inhibited, despite the normal expression pattern of proneural proteins (see below) and an expanded region of neuroectoderm. Thus, transcription factors such as FoxM1 might have key roles in neuronal differentiation during the final rounds of progenitor proliferation (Ueno et al. 2008).

### Proneural bHLH proteins

Many aspects of neurogenesis are controlled by the “master regulator” transcription factors of the bHLH proneural family such as Neurogenins (Ngns), Ascl1 and Atonal/Ath1. These act as dimers, generally with E protein partners, to control processes as diverse as cell cycle exit, neuronal differentiation and maturation, plus non-cell-autonomous progenitor maintenance through the control of the Notch-Delta pathway. The reader is directed to reviews by Bertrand et al. (2002) and Wilkinson et al. (2013). Recently, an elegant study combining both gene knock-down and overexpression has shown that Ascl1 plays a direct role in promoting progenitor proliferation in the ventral telencephalon and in NSCs in culture, through direct binding and activation of the promoters of key cell cycle progression genes such as E2F1, Skp2 and cdk2 (Castro et al. 2011). A second set of cell cycle arrest genes are also directly regulated by Ascl1 and these are preferentially activated upon overexpression of wild-type Ascl1, which concurrently induces NSCs to differentiate. Ascl1 appears to turn on only pro-proliferative targets in cycling NSCs, whereas anti-proliferative gene up-regulation occurs when Ascl1-overexpressing cells exit the cell cycle (Castro et al. 2011). The way in which Ascl1 switches between a mode promoting proliferation to one promoting differentiation is not clear, although it might involve additional DNA-binding events by components of the Notch signalling pathway at target promoters (Castro et al. 2011) or post-translational modification by cell cycle components (see below).

Given the central role of proneural bHLH proteins in neuronal differentiation, they unsurprisingly have also been implicated in cell cycle exit. In mouse embryonal carcinoma cell lines, overexpression of the proneural proteins NeuroD, Ascl1 and Ngn1 leads to the up-regulation of the cdki protein p27Kip1 (Farah et al. 2000). However, subsequent studies indicate that any up-regulation of cdkis by proneural proteins is likely to be indirect; the global transcriptional profiles of cells overexpressing proneural bHLH proteins do not identify cdkis as direct downstream targets of proneural proteins (Castro et al. 2011; Seo et al. 2007). Furthermore, consistent with coordinately regulating both differentiation and cell cycle exit, Ngn2 has recently been shown both directly to activate differentiation target genes and indirectly to repress the expression of a subset of G1-S transition cyclins, with changes being observed within 6 h of overexpression and prior to any change in levels of cdk or cdkis (Lacomme et al. 2012). The upstream transcriptional regulators that bring about gradual up-regulation of cdkis on cell cycle lengthening during the development of the CNS have yet to be fully identified.

Indeed, much data is emerging to support the idea that proneural factor activity is intimately linked with cell cycle control. In *Xenopus*, Ngn2 is required for the differentiation of primary neurons, the first neurons to differentiate from neural progenitors in the neural plate. This absolutely requires the cdki p27Xic1 and, as described above, via a mechanism independent of the cell cycle regulatory activity of p27Xic1, whereby Ngn2 protein is stabilised by p27Xic1 protein (Vernon et al 2003). This mechanism is conserved in mammalian cells (Nguyen et al. 2006b) and might also be the case for the cell-cycle-regulated stabilisation of other proneural proteins (Roark et al. 2012).

A second way that the cell cycle directly impacts on differentiation in the nervous system is by the direct post-translational modification of proneural proteins by cell cycle components. Ngn2 protein is phosphorylated on multiple Serine-Proline sites by cdks (Ali et al. 2011) and potentially other proline-directed kinases such as GSK3beta (Ma et al. 2008). This phosphorylation limits Ngn2 DNA binding, having the effect of reducing the transcription of downstream targets that drive differentiation such as NeuroD and MyT1 (Ali et al. 2011). Interestingly, the activation of the promoter of the downstream target Delta, which is required for non-cell-autonomous progenitor maintenance, is largely unaffected by Ngn2 phosphostatus (Hindley et al. 2012). This is probably because the Delta promoter is epigenetically available and therefore can tolerate reduced promoter binding seen with phosphorylated Ngn2 protein. In contrast, the NeuroD promoter requires both extensive remodelling with histone acetyl transferases and SWI/SNF-induced nucleosome repositioning (Koyano-Nakagawa et al. 1999; Seo et al. 2005a, 2005b) and so absolutely requires more avid binding by hypo-phosphorylated Ngn2 for activation.

Significantly, Ngn2 is phosphorylated on a number of Serine-Proline sites and the extent of multi-site phosphorylation is intimately linked to the level and duration of exposure to active cdk (Ali et al. 2011). Indeed, with the use of a cumulative phosphomutant series, Ali et al. (2011) have shown that mRNA output from the NeuroD promoter is quantitatively responsive to the number of Ngn2 phosphosites available (the more sites available, the lower the NeuroD expression), allowing Ngn2 to act as a “rheostat” to sense cdk level and duration. This provides a way that the cell cycle kinase environment can directly feed into the DNA-binding activity of a key inducer of differentiation; high cdk activity phosphorylates Ngn2, which is incompatible with the activation of targets driving differentiation, whereas gradual de-phosphorylation of Ngn2 in response to cell cycle lengthening over a critical threshold promotes Ngn2 DNA binding, up-regulating the transcription of specific targets that affect neuronal differentiation (Ali et al. 2011). Multi-site Serine-Proline phosphorylation of Ngn2 may have a further role in controlling progenitor maintenance versus differentiation; Notch signalling acts in the nervous system by both the inhibition of Ngn2 mRNA expression and the inhibition of the activity of the Ngn2 protein in proliferating progenitors (Bellefroid et al. 1996). Unlike the wild-type protein, phosphomutant Ngn2 protein that cannot be phosphorylated on Serine-Proline sites can induce ectopic neurogenesis, even in the presence of a constitutively active Notch intracellular domain in *Xenopus* (Hindley et al. 2012). This finding illustrates an additional way that cell cycle lengthening, resulting in decreased cdk activity, can directly lead to an enhancement of neuronal differentiation.

Many proneural proteins have a number of Serine-Proline and/or Threonine/Proline sites that might be targets of cdks. We have seen that at least Ascl1 appears to be regulated by multi-site cdk-dependent phosphorylation in a similar manner to Ngn2 and that de-phosphorylated Ascl1 has enhanced activity to promote both the onset of neuronal differentiation and aspects of neuronal maturation such as neurite outgrowth (F.R. Ali et al., in press). Whether this is a widespread mechanism of post-translational control that allows proneural proteins to respond to the cellular environment to control the balance between proliferation and differentiation throughout the CNS will be of interest.

An additional level of complexity has been added to the control of proneural protein activity by the observation that the expression of the proneural proteins Ngn2 and Ascl1 in neural progenitors is oscillatory, being controlled by a double-negative feedback loop working in anti-phase with Hes1 and Notch signalling (Imayoshi et al. 2013). This pattern changes in differentiating neurons in which proneural expression is sustained and Hes1 is repressed, although the precise mechanism for permanent repression of Hes1 is not yet clear (Shimojo et al. 2008). An innovative approach has been taken by Imayoshi et al. (2013) who have used new optogenetic techniques to modulate the frequency and amplitude of Ascl1 gene expression in Ascl1-null NSCs. Their results indicate that oscillations with a critical 3-h periodicity enhance progenitor proliferation and that an increase in the amplitude of the oscillations increases the number of proliferating cells without promoting differentiation. In contrast, sustained Ascl1 expression enhances differentiation in the Ascl1-null cells and the amplitude of expression determines the efficiency of differentiation (Imayoshi et al. 2013). This indicates that different modes of proneural protein expression result in different patterns of downstream target expression. Such “frequency modulation” of promoters has been described for the NFkappaB pathway (Paszek et al. 2010), and it will be fascinating to discover whether the same is true for the targets of proneural proteins.

## Concluding remarks

**Fig. 2:**
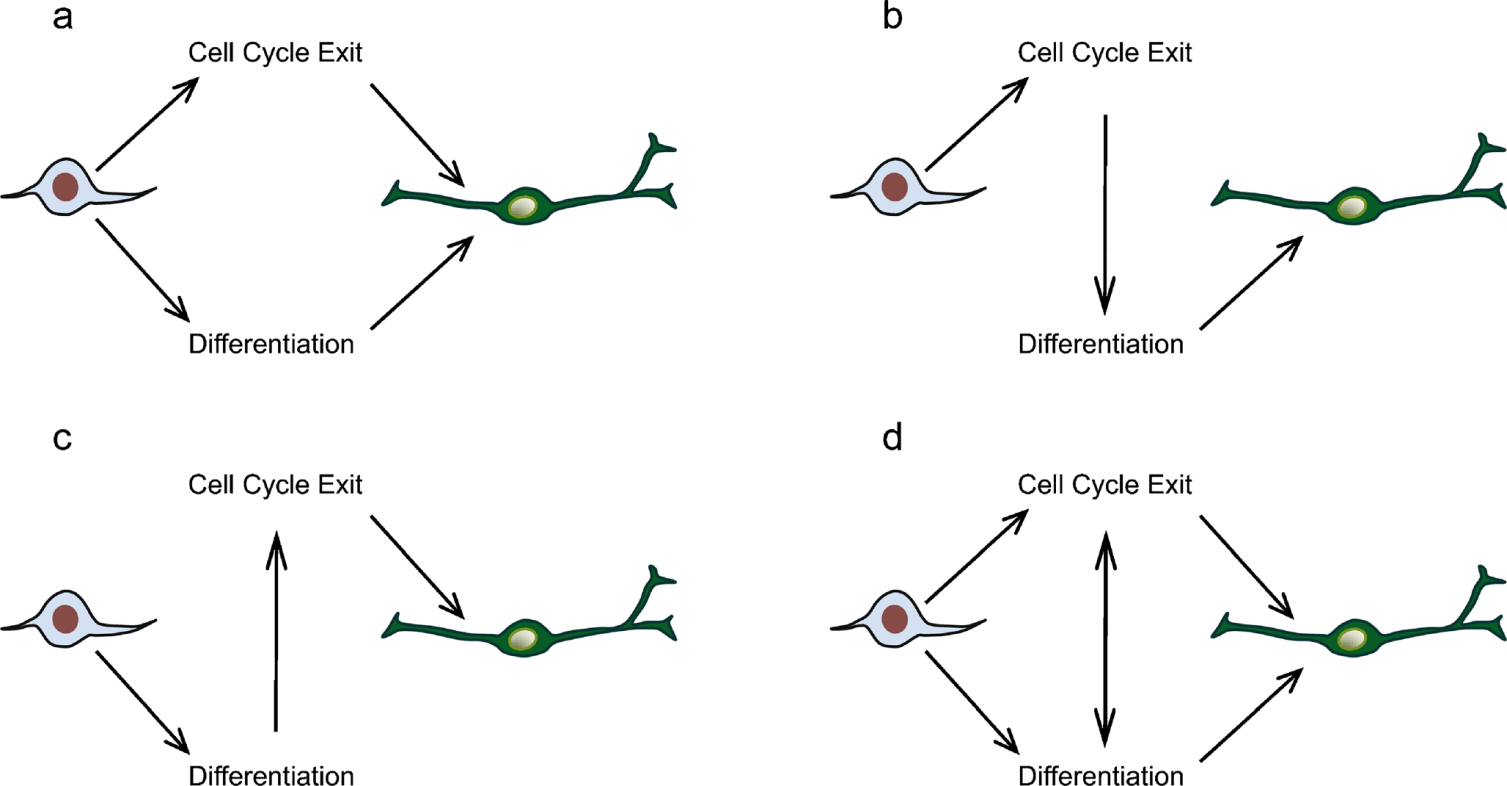
Possible modes of interaction between cell cycle lengthening/exit and differentiation in the nervous system. **a** Cell cycle and differentiation are independently regulated (e.g. Lacomme et al. 2012). **b** Induction of cell cycle lengthening and exit promotes differentiation (e.g. Calegari and Huttner 2003). **c** Initiation of differentiation results in cell cycle exit (e.g. Farah et al. 2000). **d** Cell cycle lengthening and exit are co-ordinated with differentiation by dual actions of core components of the cell cycle and differentiation machinery (e.g. Ali et al. 2011; McGarry and Kirschner 1998; Kroll et al. 1998; Vernon et al. 2003). Figure adapted from Ohnuma et al. (2001)

We have come a long way from the simple observation that cell cycle lengthening accompanies an increased propensity to undergo differentiation, moving towards a more comprehensive understanding of the molecular machinery linking the processes of cell proliferation and neuronal differentiation (Fig. 2). Connections between these processes exist at many levels, perhaps unsurprisingly as the balance between progenitor maintenance and neuronal differentiation is so critical to the generation of an appropriately patterned CNS. Emerging themes have been the realisation that single proteins, such as cdkis and Geminin, can have multiple but independent functions, sometimes separated physically within the one protein, whereas other regulators such as proneural transcription factors coordinate division and differentiation by changing their targets according to the cellular environment. Strikingly, these insights have come from a wide variety of organisms and cellular systems, highlighting the importance of using diverse models for these kinds of studies. Despite the great progress made in this area in recent years, we still have a lot to learn, with important implications for the fields of developmental biology, regenerative medicine and oncology, among others; this promises to be an exciting field over the coming decade.
